# Haplotype analysis of the *HFE* gene among populations of Northern Eurasia, in patients with metabolic disorders or stomach cancer, and in long-lived people

**DOI:** 10.1186/s12863-016-0396-z

**Published:** 2016-06-17

**Authors:** S. V. Mikhailova, V. N. Babenko, D. E. Ivanoshchuk, M. A. Gubina, V. N. Maksimov, I. G. Solovjova, M. I. Voevoda

**Affiliations:** Institute of Cytology and Genetics, Siberian Branch of Russian Academy of Sciences, Novosibirsk, Russian Federation; Institute of Internal and Preventive Medicine, Novosibirsk, Russian Federation; Novosibirsk State University, Novosibirsk, Russian Federation; Novosibirsk State Medical University, Novosibirsk, Russian Federation

**Keywords:** *HFE* gene, Haplotype analysis, Asian race, Caucasoid race, Metabolic syndrome, Type 2 diabetes mellitus, Long-lived people, Fatty liver disease, Natural selection

## Abstract

**Background:**

Previously, it was shown that the *HFE* gene (associated with human hereditary hemochromatosis) has several haplotypes of intronic polymorphisms. Some haplotype frequencies are race specific and hence can be used in phylogenetic analysis. We assumed that analysis of Caucasoid patients—living now in Western Siberia and having diseases associated with dietary habits and metabolic rate—will allow us to understand the processes of possible selection during settling of the northern part of Asia.

**Results:**

Haplotype analysis of Northern Eurasian native and recently settled ethnic groups was performed on polymorphisms rs1799945, rs1800730, rs1800562, rs2071303, rs1800708, rs1572982, rs2794719, rs807209, and rs2032451 of this gene. The CCA haplotype of the rs2071303, rs1800708, and rs1572982 was found to be associated with HLA-A2 (39 %) in Asian populations. Haplotype analysis for the rs1799945, rs1800730, rs1800562, rs2071303, rs1800708, and rs1572982 was performed on Russian patients with some metabolic disorders or stomach cancer and among long-lived people. Decreased frequencies of the TTA haplotype (T in rs2071303, T in rs1800708, and A in rs1572982) were observed in the groups of patients with diseases associated with overweight (fatty liver disease, type 2 diabetes mellitus, or metabolic syndrome + arterial hypertension) as compared with the control sample. We detected significant differences in this haplotype’s frequency between the patients with type 2 diabetes mellitus and Russian adolescents, elderly citizens, and long-lived people (χ^2^ P value = 0.003, 0.010, and 0.015, respectively).

**Conclusions:**

No significant differences in frequencies of the alleles with mutations in coding regions of the *HFE* gene (C282Y, H63D, and S65C) were detected between the analyzed patients (with stomach cancer, metabolic syndrome, fatty liver disease, or type 2 diabetes mellitus) and the control Caucasoid sample. Monophyletic origin of H63D (rs1799945) was confirmed in Caucasoids and Northern Asians. The reasons for a sharp increase in the frequency of CCA haplotype of *HFE* in the Asian race remain unclear.

**Electronic supplementary material:**

The online version of this article (doi:10.1186/s12863-016-0396-z) contains supplementary material, which is available to authorized users.

## Background

It is known that single-nucleotide polymorphisms (SNPs) of the *HFE* gene form intragenic haplotypes because this gene is located on the short arm of human chromosome 6, 4 megabases from the major histocompatibility complex (*MHC*) on the telomeric side. This human-genome locus codes for human leukocyte antigens (HLA) and is characterized by a significant linkage disequilibrium and a high polymorphism level at the same time. The recombination rate is 5-fold lower than the average in this segment of the human genome [1]. It has been shown that some *HFE* haplotype frequencies are race specific and hence can be used in phylogenetic analysis [2–5]. The common designations of the *HFE* SNPs under study and their genetic identifiers are shown in Table 1.

**Table 1:** Genetic identifiers, locations in the *HFE* gene, and common designations of the SNPs under study

SNP	Location	SNP	Location	SNP	Location
rs2794719	Intron 1	rs2071303	Intron 2, IVS2(+4)t/c	rs1800562	Exon 4, C282Y
rs1799945	Exon 2, H63D	rs2032451	Intron 3	rs1800708	Intron 4, IVS4(−44)t/c
rs1800730	Exon 2, S65C	rs807209	Intron 3	rs1572982	Intron 5, IVS5(−47)a/g

As shown previously, only 4 *HFE* intronic haplotypes for rs2071303, rs1800708, and rs1572982 (TTG, CTA, CCA, and TTA) occur in populations of Russia, each of the mutations (C282Y, H63D, and S65C) is in a linkage disequilibrium only with one of the intronic haplotype variants: TTG, CTA, and CCA, respectively [5]. Genotyping for these 6 SNPs (rs1799945, rs1800730, rs1800562, rs2071303, rs1800708, and rs1572982) in several Northern Eurasian ethnic groups revealed an increased frequency of the “youngest” and less polymorphic CCA haplotype among Asians compared to Caucasoids. Of note, South-East Asian populations also have high CCA frequency [2, 4] despite different routes of colonization in North and South Asia [6, 7]. This phenomenon may be explained either by a founder effect in an ancestral population that gave rise to the entire Asian race or by convergent selection at the beginning of (or even during) settling of Asia. The T-to-C substitution in rs1800708 is specific for this haplotype, and may change probability of formation of soluble HFE, because of its location in 1 of 4 nearby alternative donor splice sites in intron 4 of the *HFE* gene. The use of this alternative splice site leads to expression of *HFE* mRNA with retained intron 4 and production of a shortened protein formation [5]. It is known that truncated soluble HFE can associate with soluble transferrin receptor 1 [8]; in addition, soluble HFE regulates hephaestin expression [9].

It is known that excess free iron in the body leads to oxidative stress, increasing inflammation, necrosis, and apoptosis. On the other hand, iron deficiency anemias can cause immune disorders and impairment of thermoregulation and may promote carcinogenesis and psychomotor and cognitive aberrations in children [10–12]. The HFE protein is one of the key regulators of iron metabolism. It regulates capture of transferrin-bound iron from plasma and expression of the iron-regulatory hormone hepcidin [13]. Decreased expression of the *HFE* gene or misfolded HFE protein can increase iron transport from enterocytes to the bloodstream and iron uptake by all cells possessing transferrin receptors (especially by hepatocytes). In combination with increased dietary intake of iron, the above condition can cause iron overload and then hemochromatosis, which is mainly characterized by iron deposition in parenchymal tissues, hepatomegaly, cirrhosis, diabetes mellitus, and skin hyperpigmentation. C282Y/C282Y genotype carriers constitute up to 100 % of patients with hereditary hemochromatosis. Nonetheless, clinical penetrance of hereditary hemochromatosis among C282Y homozygotes is incomplete in human populations and depends on other genetic and environmental factors [14]. In addition, it was shown that frequencies of C282Y and H63D alleles are increased among patients with various cancers and metabolic disturbances; in particular, the H63D mutation of the *HFE* gene alters cholesterol metabolism [15]. This phenomenon is usually explained by adverse effects of oxidative stress and immune disorders in carriers of these mutations [16]. Nevertheless, the presence of the same mutations can provide a protective effect against anemia in geographical regions with frequent iron deficiency [17].

During settlement of human populations across the planet, these populations underwent many bottlenecks with reductions in size and large migrations of people. Migration out of Africa, settling of new climatic zones, domestication of animals, and the agricultural revolution caused dietary changes necessary to adapt to new pathogens and new climatic conditions and possibly to develop cognitive abilities [18, 19]. The continental climate of Northern Asia resulted in human adaptation to low humidity; frequent fluctuations in atmospheric pressure, temperature, and insolation; and to reduced availability of plant food. During the spread of humans across Asia, selection for certain adaptive traits could have been one of the reasons for formation of the Asian race.

We assumed that haplotype analysis in Caucasoid patients—living now in Western Siberia and having diseases associated with dietary habits and metabolic rate—will allow us to understand the processes of possible selection during settling of the northern part of Asia.

Patients with various lipid metabolism disorders were selected because lipid metabolism strongly affects energy balance, capacity for cold adaptation, and survival during long-term nutritional restriction. In particular, patients with a combination of metabolic syndrome and arterial hypertension were genotyped here because it is believed that predisposition to hypertension in humans is a consequence of maladaptation during settling of cold climatic zones [18].

*Metabolic syndrome* (MS) is a complex metabolic disorder that increases the risk of cardiovascular disease and diabetes. MS’s main diagnostic feature is abdominal obesity; in addition, patients may have high blood pressure, elevated plasma levels of triglycerides, and low levels of high-density lipoprotein cholesterol and fasting plasma glucose in various combinations. MS patients show elevated levels of systemic inflammation markers and hepcidin [20, 21].

*Fatty liver disease* (FLD) is a liver disorder that is characterized by fat accumulation in hepatocytes. It can proceed with or without the effects of insulin resistance [22].

*Type 2 diabetes mellitus* (T2DM) constitutes 85–90 % of all cases of diabetes and is associated with obesity or overweight in most cases. It is characterized by hyperglycemia in the context of insulin resistance and a relative deficit of insulin [21].

To estimate the possible influence of different *HFE* alleles on the development of cancer associated largely with cooking methods and dietary habits, a group of patients with stomach cancer was genotyped here.

*Stomach cancer* is a malignant tumor developing in the gastric epithelium. The main risk factors of stomach cancer are *Helicobacter pylori* infection, smoking, and genetic predisposition. Inappropriate dietary habits such as consumption of high-salt foods, fried or stewed creatinine-containing foods (e.g., red meats), low consumption of fruit and vegetables, and iron deficiency anemia are additional risk factors [11, 23].

In this study, we analyzed the distribution of intragenic haplotypes and genotypes of the *HFE* gene i) among recently settled and indigenous Northern Eurasian populations and ii) in patients with metabolic disorders and among long-lived people. We tested the hypothesis that some *HFE* gene alleles were under positive selection pressure or hitchhiked with some *HLA-A* haplotype under specific environmental conditions of Northern Eurasia.

## Results and discussion

### HFE haplotype analysis

**Fig. 1:**
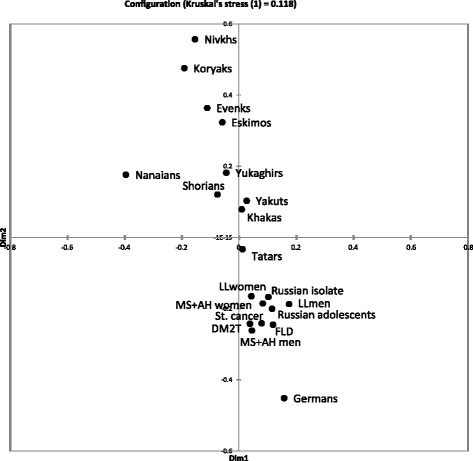
A multidimensional scaling plot of distances among populations and groups of patients on the basis of Pearson’s matrix (Additional file 1: Table S1). MS + AH: metabolic syndrome associated with arterial hypertension, T2DM: type 2 diabetes mellitus, FLD: fatty liver disease, LL: long-lived people

The genotyping data on rs1799945 (H63D), rs1800730 (S65C), rs1800562 (C282Y), rs2071303, rs1800708, and rs1572982 of the *HFE* gene in Siberian populations and in patients are summarized in Table 2. These data were used for construction of Pearson’s matrix (Additional file 1: Table S1) and a multidimensional scaling (MDS) plot of genetic similarities (Fig. 1).

**Table 2:** The genotyping data on rs1799945 (H63D), rs1800730 (S65C), rs1800562 (C282Y), rs2071303, rs1800708, and rs1572982 of the *HFE* gene in Northern Asian populations and in some groups of patients. MS + AG: metabolic syndrome associated with arterial hypertension, T2DM: type 2 diabetes mellitus

Exon SNPs	Intron SNPs	Caucasoids			Patients						Long-lived people			Tatars	Asians								
		Russian adolescents	Russian old believers (an isolate)	Germans	MS + AH total	MS + AH men	MS + AH women	Fatty liver disease	T2DM	Stomach cancer	total	men	women		Shorians	Khakas	Yukaghirs	Koryaks	Nivkhs	Evenks	Nanaians	Yakuts	Eskimos
C282Y/C282Y	TTG/TTG	0	0	0	0	0	0	1	0	0	0	0	0	0	0	0	0	0	0	0	0	0	0
H63D/H63D	CTA/CTA	10	0	1	1	0	1	1	4	5	7	1	6	1	0	0	0	0	0	0	0	0	0
H63D/S65C	CTA/CCA	1	0	0	1	1	0	0	0	0	0	0	0	1	0	1	0	0	0	0	0	0	0
C282Y/H63D	TTG/CTA	4	0	1	1	1	0	1	1	2	1	0	1	0	0	1	0	0	0	0	0	0	0
C282Y/WT	TTG/TTG	9	3	3	10	5	5	2	3	1	3	1	2	0	0	0	4	0	0	0	0	0	0
	TTG/TTA	3	0	0	7	1	6	0	0	1	2	1	1	0	1	0	0	0	0	0	0	0	0
	TTG/CTA	0	2	0	2	2	0	1	2	2	1	0	1	0	0	0	0	0	0	0	0	0	0
	TTG/CCA	0	2	0	1	0	1	0	0	0	2	0	2	0	0	0	1	0	0	0	0	0	0
H63D/WT	CTA/CCA	11	0	1	10	0	10	1	1	2	8	1	7	1	8	0	4	2	3	0	0	0	0
	CTA/CTA	18	4	3	5	3	2	3	6	3	7	1	6	0	1	1	0	1	0	0	1	0	0
	CTA/TTA	12	4	5	14	5	9	3	6	2	4	1	3	4	5	1	0	0	0	0	0	1	0
	CTA/TTG	52	13	16	50	19	31	7	22	21	29	8	21	12	6	5	3	0	0	2	4	0	0
S65C/WT	CCA/CTA	2	1	0	1	1	0	0	0	0	0	0	0	0	1	2	0	0	0	0	0	0	0
	CCA/CCA	0	1	1	0	0	0	0	1	0	0	0	0	0	1	0	1	1	0	0	0	0	0
	CCA/TTG	5	0	3	6	4	2	4	4	2	1	0	1	5	1	0	0	0	0	0	0	0	0
	CCA/TTA	2	2	0	1	1	0	1	1	0	1	0	1	1	2	1	1	0	0	0	0	0	0
WT/WT	TTG/TTG	77	26	14	104	39	65	19	54	44	75	12	63	54	28	17	18	5	2	5	5	9	6
	TTG/TTA	54	18	7	46	10	36	11	12	21	33	13	20	26	10	11	5	2	0	3	0	5	5
	CCA/CCA	3	1	0	6	3	3	1	2	2	2	0	2	5	11	8	10	17	9	10	2	2	8
	CCA/CTA	5	3	0	5	2	3	0	1	6	1	0	1	11	12	2	1	7	1	1	9	0	2
	CTA/CTA	4	0	2	2	1	1	0	1	5	1	0	1	2	2	2	0	0	0	0	2	0	0
	CTA/TTA	16	2	1	10	1	9	0	5	5	9	2	7	7	4	2	2	0	0	0	0	0	1
	TTA/TTA	4	2	0	8	4	4	0	1	3	4	0	4	5	6	4	0	1	0	0	0	0	3
	TTG/CTA	27	6	19	39	19	20	11	15	13	27	6	21	14	5	3	3	4	0	2	0	1	1
	CCA/TTA	7	3	0	11	2	9	2	2	3	9	2	7	10	9	10	7	4	4	1	1	5	5
	TTG/CCA	30	9	3	28	9	19	5	11	7	27	5	22	37	29	14	21	37	10	14	9	8	11
Total		356	102	80	369	133	236	74	155	150	254	54	200	196	142	85	81	81	29	38	33	31	42

There is a correlation between genetic distances in Fig. 1 and geographic locations of the Asian populations analyzed. Nanaians, Nivkhs, Koryaks, Evenks, Eskimos, and Yukaghirs inhabit the eastern part of Northern Asia. Shorians, Khakas, Yakuts, and Siberian Tatars are referred to as Turkic peoples. Shorians, Khakas, and Tatars are the inhabitants of Western Siberia; Yakuts migrated to their present geographic area in the Sakha (Yakutia) Republic from South Siberia in the 13th century. Siberian Tatars are characterized by intermediate anthropological and genetic features between Caucasoids and Asians. Ethnic Russians began to spread across Siberia at the end of the 19th century; Germans were resettled to South Siberia from the European part of Russia in the 20th century. Populations of Eastern Siberia and Altai Germans (they originated in Western Europe) are the most genetically distant groups. Turkic peoples (including Tatars) and ethnic Russians are between them in the plot. Our groups of patients lie near the ethnic Russian population on the MDS plot (Fig. 1).

The *HFE* haplotype frequencies for rs1799945 (H63D), rs1800730 (S65C), rs1800562 (C282Y), rs2071303, rs1800708, and rs1572982 in the groups under study are summarized in Table 3.

**Table 3:** Amounts of *HFE* haplotypes and their frequencies for rs2071303, rs1800708, rs1572982, C282Y, H63D, and S65C in various populations, groups of patients, and long-lived people

Groups	TTG WT	TTG C282Y	CTA WT	CTA H63D	TTA	CCA WT	CCA S65C
Populations							
Russian adolescents	331	16	75	118	103	59	10
	0.46	0.02	0.11	0.17	0.14	0.08	0.01
Russian old believers (an isolate)	101	7	18	21	33	20	4
	0.5	0.03	0.09	0.1	0.16	0.1	0.02
Germans, Altai	79^a^	4	26	29	13	5	4
	0.49	0.03	0.16	0.18	0.08	0.03	0.03
Siberian Tatars	207	0	34	21	56	71	7
	0.52		0.09	0.05	0.14	0.18	0.02
Shorians	107^a^	1	26	20	44	81	5
	0.38	0.004	0.09	0.07	0.15	0.29	0.02
Khakas	67	1	13^a^	9	33	43	4
	0.39	0.006	0.08	0.05	0.19	0.26	0.02
Yukaghirs	71	5	8	7	14	55	2
	0.44	0.03	0.05	0.04	0.09	0.34	0.01
Yakuts	32	0	1	1	11	17	0
	0.52		0.02	0.02	0.18	0.27	
Nivkhs	15	0	1	3	4	35	0
	0.26		0.02	0.05	0.07	0.6	
Koryaks	53	0	12	3	8	86^a^	0
	0.33		0.07	0.02	0.05	0.53	
Evenks	30	0	3	2	4	37	0
	0.39		0.04	0.03	0.05	0.49	
Nanaians	23	0	14	5	1	23	0
	0.35		0.21	0.08	0.02	0.35	
Eskimos	29	0	4	0	17	34	0
	0.35		0.05		0.2	0.4	
Patients, Novosibirsk							
MS + AG total	387	21	66	84	105	68	9
	0.52	0.03	0.09	0.11	0.14	0.09	0.01
MS + AH, women	243	12	36	54	77	48	2
	0.51	0.03	0.08	0.11	0.16	0.1	0.004
MS + AH, men	144	9	30	28	28	20	7
	0.54	0.03	0.11	0.11	0.11	0.08	0.03
Fatty liver disease	78	6	15	17	17	10	5
	0.53	0.04	0.1	0.11	0.11	0.07	0.03
T2DM	172	6	32	46	28	20	6
	0.55	0.02	0.1	0.15	0.09	0.06	0.02
Stomach cancer	154	6	38	40	38	22	2
	0.51	0.02	0.13	0.13	0.13	0.07	0.01
Long-lived people							
total	270	9	47	63	66	51	2
	0.53	0.02	0.09	0.12	0.13	0.1	0.004
men	57	2	9	13	19	8	0
	0.53	0.02	0.08	0.11	0.18	0.07	
women	213	7	38	50	47	43	2
	0.53	0.02	0.1	0.13	0.12	0.11	0.005

WT are gene variants without the exonic SNPs; MS + AH: metabolic syndrome associated with arterial hypertension, T2DM: type 2 diabetes mellitus, ^a^deviations from Hardy-Weinberg equilibrium

The genotype distribution in all populations is in Hardy-Weinberg equilibrium with the exception of Khakas for CTA WT (χ^2^ P value 0.021), Germans and Shorians for TTG WT (χ^2^ P value 0.0138 and 0.0051, respectively), and Koryaks for CCA WT (χ^2^ P value 0.023). The deviation from Hardy-Weinberg equilibrium in the Khakas group is probably caused by low frequency of the CTA WT allele and small sample size. Germans, Shorians, and Koryaks inhabit isolated ethnic settlements, with the possibility of marriages between relatives. This state of affairs can explain the Hardy-Weinberg deviations in these groups.

### HLA-typing of the CCA haplotype homozygotes for rs2071303, rs1800708, and rs1572982

In Table 3, a west–east gradient of the CCA WT haplotype’s frequency can be seen among Asian populations. East Asian Evenks, Koryaks, and Eskimos have a higher CCAWT frequency than do Central Asian Tartars, Shorians, Khakas, and Yakuts. The reason for formation of such a gradient may be a genetic drift because it is known that geographic distance to East Africa correlates with the loss of genetic and phenotypic heterogeneity [6, 24, 25]. Besides, an increase in the CCA haplotype frequency may be possible both because of hitchhiking and as a result of positive selection owing to possible changes in regulation of *HFE* expression. This is because change in gene expression drives human adaptation 10-fold more likely than changes in amino acid sequence [26]. The most likely candidates for hitchhiking are *HLA* class I genes. It was proven that they are under strong selection pressure [25]. A model of pathogen-driven balancing selection implies maintenance of maximal heterozygosity of the *HLA* locus in populations, although it has been demonstrated that the number of *HLA* alleles depends on a pathogen’s (especially a virus’s) richness in a certain area [27, 28].

*HLA* typing is a widespread method for haplotype analysis of the *MHC* locus. Some *HFE* alleles were found to be in linkage disequilibrium with certain *HLA* haplotypes. The C282Y, H63D, and S65C mutations of *HFE* are associated with alleles *HLA-A3* (43–75 %), *HLA-A29* (16–27 %), and *HLA-A26* of the *HLA-A* locus, respectively [29–31]. Many of *HLA* haplotypes of *MHC* were found to be associated with autoimmune and metabolic disturbances and with some infectious and oncological diseases [32]. Selection for a certain *HLA* haplotype during the settling of Asia may be one of the reasons for the growth of the number of CCA WT haplotype carriers among Asians. *HLA-A*, −*B*, and *-C* alleles were identified in 18 homozygous CCA WT carriers. The results of HLA typing are summarized in Table 4.

**Table 4:** HLA genotypes in CCA/CCA haplotype carriers

Ethnic groups	HLA-A	HLA-B	HLA-C
Russians	A1/A3	B35/B58	Cw4/Cw7
	A3/A3	B35/B35	Cw4/Cw4
Tatars	A2/A24	ND	Cw2/Cw8
	A11/A24	B60/ND	Cw9/Cw9
Tuvinians	A2/A2	B61//ND	Cw6/Cw8
	A2/A11	B46/B60	Cw1/Cw10
Shorians	A2/A24	B56/B61	Cw1/Cw6
	A3/A31	B7/B41	Cw8/Cw8
Khakas	A2/A24	ND	Cw10/Cw10
	A2/A24	B60/ND	Cw1/Cw10
Yakuts	A2/A24	B61/B61	Cw10/Cw10
	A31/A31	ND	Cw9/Cw9
Koryaks	A2/A31	B27/B61	Cw2/Cw10
	A2/A24	B48/B48	Cw7/Cw8
Altaian	A1/A2	B44/B6	Cw5/Cw7
Kazakh	A2/A80	ND	Cw7/Cw8
Evenks	A3/A3	B35/B35	Cw4/Cw4
	A31/A31	B21/B48	Cw8/Cw10

*ND* not determined

In the Asian sample (28 chromosomes analyzed), CCA was most frequently associated with *HLA-*A2 (39 %). A higher-resolution kit is required for identification of alleles A2 and A210 (A*02010101-02/04-22/24/26/33/36/37/39/40/42-49/51-55/57/61/63/64/66-72/7401-77/79/81-97/99 and A*9201/02/04/06-09, respectively), which yielded identical results here. In addition, CCA haplotype was detected in combination with *HLA-*A1, A3, A11, A24, A31, and A80 (1, 3, 1, 4, 5, and 1 chromosome, respectively). Because *HLA-B* and *HLA-C* are located farther from *HFE* on the chromosome than *HLA-A* is, they do not have similar statistical associations. Selection for the CCA haplotype among Asians is hardly caused by its linkage to *HLA-*A2 because the latter is one of the most widespread in Asians, Tatars, and Caucasoids [33–35].

### TTG haplotype analysis

It is known that the TTG WT haplotype (T in rs2071303, T in rs1800708, and G in rs1572982) has 3 subtypes, one of them includes 3 linked mutations in intron 3 of the *HFE* gene, and another includes 5 linked mutations in the 5′ untranslated region (5′UTR) and in intron 1 [4]. For detection of a variety of TTG WT haplotypes, a search for T in rs807209 and G in rs807209 was carried out. Thirteen Russians, 9 Yakuts, 25 Shorians, 9 Khakas, 2 Kazakhs, and 35 Tatars homozygous for the TTG WT allele were analyzed for rs807209 because it was previously detected in Africans [4]. T in rs807209 was not found in these 186 chromosomes. Rather, the results showed a lack of haplotypes containing it among Caucasoid and Northern Asian populations at appreciable frequencies.

Another TTG haplotype with 5 linked mutations in the 5′UTR and intron 1 seems to be the second most common among all Europeans. It is in this haplotype that the C282Y mutation arose. Additionally, the same combination of polymorphisms was found in the CTA + H63D and all CCA haplotypes. It is believed that these identical mutations arose in different haplotypes via recombination events [4]. A total of 163 chromosomes (103 ethnic Russian and 60 Asian chromosomes, including 20 Khakas, 12 Eskimos, 4 Nivkhs, 8 Nanaians, and 16 Chukchis) carrying the TTG WT haplotype were analyzed for rs2794719 in intron 1 of *HFE.* The frequency of G in rs2794719 among TTG WT chromosomes was 0.38 in ethnic Russians and 0.13–0.42 in Asians.

Thus, there is no significant difference in frequencies of 3 known subtypes of the TTG haplotypes between Asian and Caucasoid TTG WT chromosomes in the Northern part of Eurasia.

### CTA haplotype analysis

Chromosomes carrying the H63D mutation were analyzed for rs2032451. This SNP occurs among Europeans with the frequency of ~0.17 according to data at http://www.ncbi.nlm.nih.gov. The genotyping results are presented in Table 5.

**Table 5:** The rs2032451 polymorphism among H63D carriers

Ethnic groups	H63D+/+	Number	H63D+/−	Number	H63D−/−	Number
	rs2032451		rs2032451		rs2032451	
Russians	T/T	3	G/T	9	G/G	8
Tatars	T/T	1	G/T	18		
Mansis			G/T	5		
Nanaians			G/T	5	G/G	6
Nivkhs			G/T	3	G/G	5
Altaians	T/T	1	G/T	4		
Khakas			G/T	9		
Koryaks			G/T	3		
Kazakhs			G/T	9		
Shorians			G/T	16		
Chukchis			G/T	2		
Total		5		83		19

All 5 H63D homozygotes had T/T in rs2032451; all 83 H63D heterozygotes had G/T. In carriers of other genotypes, no T was detected in rs2032451. On the other hand, verification of possible presence of this SNP in some other haplotypes requires bigger cohorts. These findings confirmed the data on a linkage between rs1799945 and rs2032451 that were previously obtained in a small sample [4]. The H63D mutation seems to be associated with T in rs2032451 in addition to the previously described CTA haplotype for rs2071303, rs1800708, and rs1572982 of the *HFE* gene in most of human populations of Eurasia.

Thus, it was confirmed that the maximal difference in allele frequency between Asians and Caucasoids is observed in the CCA haplotype of *HFE* (3–8 % vs. 27–60 %). Siberian Tatars show an intermediate frequency (18 %). Furthermore, there is a significant difference in the frequency of the CTA haplotype containing the H63D mutation (10–18 % among Caucasoids vs. 0–8 % among Asians). Frequencies of other detected haplotypes showed ethnic differences comparable with interracial ones. This finding allowed us to hypothesize a selection in rs1800708 and rs1799945 during the split of the Asian race and Caucasian race. The HFE protein with H63D fails to induce hepcidin expression via the bone-morphogenic-protein pathway [36]. It is known that H63D is associated with several neurodegenerative diseases and porphyria cutanea tarda [37–39]. The reasons for the widespread occurrence of H63D in European populations are not clear. Because monophyletic origin of H63D was proven in Caucasoids and North Eurasian Asians, it can be supposed that H63D was under negative selection pressure in Asia. No changes were demonstrated in the structure of the HFE protein of the CCA haplotype without the S65C mutation. On the other hand, we previously found that C in rs1800708 (which is crucial for this haplotype) is located in a potential alternative donor splice site and may alter the probability of production of a soluble HFE protein [5].

### Genetic analysis of the patients and long-lived people

The results on genotyping of patients with MS, T2DM, FLD, or stomach cancer and long-lived people are summarized in Table 3 too. As mentioned above, patients with metabolic disturbances associated with overweight or obesity tend to have iron metabolism disorders. The increased serum ferritin and hepcidin levels are observed in MS subjects [40]. Patients with 4 or more signs of MS show more than a 4-fold increase in concentration of hepcidin (iron regulatory hormone). Normally, its concentration can increase in response to an infection and causes restriction of iron release from the cells into the bloodstream. This change inhibits proliferation of viruses and bacteria but can cause anemia of chronic diseases. Such a metabolic change is expected to worsen insulin resistance and promote cardiovascular complications in patients with MS. Apparently, a mutation in the *HFE* gene, which is a hepcidin regulator, can affect both the probability of MS development and the course of the disorder [40]. An association between the H63D mutation of *HFE* and essential hypertension was described in a Finnish cohort [41]. Data on the association of *HFE* and predisposition to T2DM and/or its complications are different depending on a population. Most likely, the effect of the *HFE* mutations is race specific [42]. Patients with FLD show increased H63D frequency in Korea [43]. In contrast, we observed no significant differences between patients and the control in frequencies of alleles C282Y, H63D, and S65C. Thus, the presence of these mutations has no effect on predisposition to AH, FLD, T2DM, or stomach cancer in Novosibirsk patients. Among the latter, however, more detailed tumor typing is required, so that risk factors (including dietary habits) can be specifically attributed to different subtypes [23]. The observed lack of a difference between the patients and the general population in frequencies of alleles C282Y, H63D, and S65C can be caused by a widespread iron deficiency anemia in the residents of Siberia [44]. This anemia may also attenuate the effect of the low-penetrance mutations on a phenotype. Frequency of the H63D allele in the adolescent sample, however, was higher than previously reported for adult Russians in Novosibirsk city and for the European part of Russia (0.17 vs. 0.13 and 0.15, respectively) [5, 45]. This frequency was found to be significantly different from the frequency in the sample of long-lived people (*P* = 0.016). In a comparison of long-lived people with adult population groups in Novosibirsk city, a decrease in C282Y frequency was noted among long-lived people [46]. We found no such difference in the frequency of C282Y between long-lived people and adolescents. The differences in frequencies of the *HFE* alleles—as a function of average age in a group—require further analysis.

We did not detect a difference in the CCA WT allele frequency between any populations and groups of patients among Russians (Table 3). It is possible that selection for specific variants in this locus may facilitate adaptation to infections and parasites because adaptation to pathogens is considered the most significant during human evolution [10, 19, 47].

Our results revealed that frequencies of the TTA haplotype of rs2071303, rs1800708, and rs1572982 of *HFE* in all groups of patients whose disorders are associated with overweight or obesity (with the exception of MS + AH women) are lower than those in the groups of adolescents, elderly people (old-believers, average age 70), and long-lived men. Significant differences in this haplotype’s frequency were found between the T2DM sample and the groups of adolescents, elderly citizens, and long-lived people (*P* = 0.003, 0.01, and 0.015, respectively). Nonetheless, the TTA haplotype’s frequency in the groups of Russian patients was close to its frequency in the Altai German population (Table 3). This result shows variability of the TTA frequency among Caucasoids and, possibly, its ethnic specificity among the patients. Most likely, the observed difference in the TTA frequency is not due to differences in iron metabolism. It is possible that one of common subtypes of the TTA haplotype is linked to *MHC* genes or any other nearby genes that have protective effects against overweight or some obesity-related complications. The TTA haplotype frequencies differ significantly between men and women in MS + AH groups (P = 0.047). Elucidation of the reasons for these genetic differences requires a more detailed study. The MS + AH female sample may be more genetically heterogeneous than the male one.

## Conclusions

The reasons for a sharp increase in the frequency of the CCA haplotype of the *HFE* gene in the Asian race remain unknown. In 39 % of cases, this haplotype is linked with HLA-A2 in Asians, but HLA-A2 is a common *HLA-A* allele everywhere. It is not clear whether the observed gradient of the CCA WT frequency is a consequence of genetic drift on the territory of Northern Eurasia or a result of selection for this allele, which is characterized by some additional capabilities of gene expression regulation. Perhaps analysis of patients with infectious diseases that are widespread in the region will allow researchers to answer this question. Frequencies of the TTA haplotype of the *HFE* gene—in the groups of patients whose disorders are associated with overweight—are lower than such frequencies in Russian populations. This topic requires a more detailed study. No significant differences in frequencies of the alleles with mutations in coding regions of *HFE* (C282Y, H63D, and S65C) were detected between the analyzed groups of patients (with stomach cancer, metabolic syndrome, fatty liver disease, or type 2 diabetes mellitus) and the control sample. Monophyletic origin of H63D (rs1799945) was confirmed in Caucasoids and Northern Asians.

## Methods

### Populations studied

The control adolescent sample (356 individuals) aged 14–17 years of both sexes was selected from the inhabitants of Novosibirsk city. The German sample was drawn from the inhabitants of a German settlement in Altai region (Veseloyarsk settlement) and consists of 80 individuals. The Siberian Tatar sample (196 people) was selected in the Novosibirsk region, Chanovsky district. Tatars are a Turkic people, which have intermediate anthropological and genetic features between Caucasoids and Asians. Native Asian groups were selected from Western Asian peoples, including Shorians (142 individuals, Kemerovo region, Tashtagol district), Khakas (85 individuals, Republic of Khakassia, Abakan town), Yakuts (31 individuals Sakha [Yakutia] Republic, Namtsy settlement), and from Eastern Asian peoples, including Yukaghirs (81 individuals, Sakha [Yakutia] Republic, Verhnekolymsky district, Nelemnoe settlement), Evenks (38 individuals, Amur region, Tyndinsky district), Nivkhs (29 individuals, Sakhalin region, Noglinsky district), Koryaks (81 individuals, Kamchatka Krai, Ossora settlement), Eskimos (42 individuals, Chukotka Autonomous Okrug, Chukotsky district), and Nanaians (33 individuals, Khabarovsk region). In addition, we analyzed the sample of Russian old believers (102 individuals), average age 70. This population chose to live in isolation since the 17th century in Kurgan region (South Ural).

The groups of Altaians and Kazakhs (Altai Republic, Kosh-Agach region), Chukchi (Chukotka Autonomous Okrug), and Mansi (Finno-Ugric people from the basin of the Ob river) that were previously genotyped for SNPs rs1799945, rs1800730, rs1800562, rs2071303, rs1800708, and rs1572982 [5] were partly used in the analysis for SNPs rs2032451, rs2794719, and rs807209.

Ethnicity of individuals was identified using questionnaires and additional cross-examination with elucidation of a nationality of ancestors (at least in 3 generations).

### Groups of patients

The sample of patients with MS consisted of 236 women (average age 59, mean body-mass index [BMI] 34) and 133 men (average age 58, mean BMI 30). In this study, only patients with arterial hypertension (systolic arterial pressure greater than 140 mmHg and diastolic one greater than 90 mmHg) were enrolled. The sample was drawn randomly from the cohort of Caucasoid inhabitants of the Oktyabr’skii district of Novosibirsk city (age 45–69 years), which were found during the HAPIEE project (Health, Alcohol, and Psychosocial factors In Eastern Europe) [48]. The T2DM sample consisted of 68 women and 87 men (average age 59, mean BMI among males: 31, females: 32). The sample was randomly selected from the HAPIEE. Individuals were assumed to have T2DM if they had a glucose level above 11 mM after a 2-hour oral glucose tolerance test. The sample of patients with FLD included 74 individuals from Novosibirsk city (men’s average age 47 and mean BMI 33; women’s average age 54 and mean BMI 35). The sample of patients with stomach cancer was drawn from the patients of Novosibirsk city and included 150 patients (men and women equally) who had various histological types of stomach cancer (the average age of males was 56, females 58). All patients had a physician-confirmed diagnosis. The sample of long-lived people consisted of 200 women and 54 men aged 90–105 years from cities Novosibirsk, Tomsk, and Tumen.

No differences between men and women were observed in the *HFE* allele frequencies in groups stomach cancer, T2DM, and FLD; the same was true for the adolescent and old-believer groups; therefore, the two genders were combined there.

### Genetic analysis

The group analysis for rs1799945, rs1800730, rs1800562, rs2071303, rs1800708, and rs1572982 and identification of *HFE* haplotypes were performed by PCR with analysis of restriction fragment length polymorphism as described elsewhere [5]; 2 298 individuals were analyzed. The analysis for rs2032451 in intron 3 of *HFE* was performed by PCR-RFLP using primers 5′-GGCCTCACTTGATATTTTGTCCTGG-3′ and 5′-GGCCCTTGCTTTTTATTTAACC-3′ and the BsuRI restriction endonuclease. Analysis for rs2794719 in intron 1 of *HFE* was performed by PCR-RFLP using primers 5′-TCCTGGCAAATTTATTCAATGGTCC-3′ and 5′-GTTTCCCTGCTCTCTCCCTTG-3′ and the BssEcI restriction endonuclease. Analysis for rs807209 in intron 3 of *HFE* was performed using a 7900HT StepOnePlus Real-Time PCR System and TaqMan® SNP Genotyping Assays (Applied Biosystems, USA).

### HFE haplotype analysis

As shown previously, only 4 HFE intronic haplotypes of rs2071303, rs1800708, and rs1572982 (TTG, CTA, CCA, and TTA) occur in populations of Russia; each of the mutations (C282Y, H63D, and S65C) was in linkage disequilibrium only with one of the intronic haplotype variants: TTG, CTA, and CCA, respectively [5]. This situation allowed us to identify intragenic *HFE* haplotypes by analyzing the groups for rs1799945, rs1800730, rs1800562, rs2071303, rs1800708, and rs1572982.

### HLA typing

HLA typing was performed using the HLA-ABC Low Res Kit (Invitrogen, USA). Eighteen homozygous carriers of the CCA intragenic haplotype without the S65C mutation were thus analyzed, including 2 ethnic Russians, 2 Siberian Tatars, 2 Evenks, 2 Khakas, 2 Shorians, 2 Koryaks, 2 Yakuts, 2 Tuvinians, 1 Altaian, and 1 Kazakh (the last 4 individuals are from previously genotyped groups of Central Asian peoples [5]).

### Statistical analysis

The populations were tested for Hardy-Weinberg equilibrium by Pearson’s χ^2^ test. Haplotypic frequencies were compared between the studied groups by χ^2^ test in the SPSS 16.0 software. A difference between two groups was considered significant at a *P* value <0.05.

An MDS plot was constructed on the basis of Pearson’s pairwise correlation matrix between the genotype frequency profiles (Additional file 1: Table S1). The XLStat package (www.xlstat.com) was used to produce both Pearson’s correlation matrix and the MDS plot. For MDS, the absolute values of Pearson’s correlation were used. The target minimization function was Kruskal’s stress statistic; initial configuration was set to “random”; the number of repetitions was 5; the convergence threshold was set to 0.00001, with the maximal number of iterations 500.

## Abbreviations

BMI, body-mass index; FLD, fatty liver disease; HLA, human leukocyte antigen; MDS, multidimensional scaling; MHC, major histocompatibility complex; MS, metabolic syndrome; SNP, single-nucleotide polymorphism.

## Additional file

Additional file 1: Table S1. Pearson’s correlation matrix between *HFE* genotype frequency profiles. Values in bold are different from 0 with a significance level alpha = 0.05. MS + AH: metabolic syndrome associated with arterial hypertension, T2DM: type 2 diabetes mellitus, FLD: fatty liver disease, St. cancer: stomach cancer, LL: long lived. (XLSX 13 kb)
